# Parasite Cystatin: Immunomodulatory Molecule with Therapeutic Activity against Immune Mediated Disorders

**DOI:** 10.3390/pathogens9060431

**Published:** 2020-05-30

**Authors:** Vishal Khatri, Nikhil Chauhan, Ramaswamy Kalyanasundaram

**Affiliations:** Department of Biomedical Sciences, University of Illinois College of Medicine, Rockford, IL 61107, USA; nikhilc3@uic.edu (N.C.); ramswamy@uic.edu (R.K.)

**Keywords:** helminth therapy, cystatin, chronic inflammatory disorders, autoimmune disorders, biotherapeutic

## Abstract

The use of parasites or their products for treating chronic inflammation associated diseases (CIADs) has generated significant attention recently. Findings from basic and clinical research have provided valuable information on strengthening the notion that parasites’ molecules can be developed as biotherapeutic agents. Completion of the genome, secreotome, and proteome of the parasites has provided an excellent platform for screening and identifying several host immunomodulatory molecules from the parasites and evaluate their therapeutic potential for CIADs. One of the widely studied host immunomodulatory molecules of the parasites is the cysteine protease inhibitor (cystatin), which is primarily secreted by the parasites to evade host immune responses. In this review, we have attempted to summarize the findings to date on the use of helminth parasite-derived cystatin as a therapeutic agent against CIADs. Although several studies suggest a role for alternatively activated macrophages, other regulatory cells, and immunosuppressive molecules, in this immunoregulatory activity of the parasite-derived cystatin, there is still no clear demonstration as to how cystatin induces its anti-inflammatory effect in suppressing CIADs.

## 1. Introduction

Incidences of chronic inflammation associated diseases (CIADs) are surging worldwide [1]. CIADs include, but not limited to, inflammatory bowel disease, multiple sclerosis, rheumatoid arthritis, and allergies. The mainstream therapy for CIADs is not advancing enough. In addition, such therapy is suppressive with limited efficacy and short-term effects, may lead to cancer or other opportunistic infections, and is often costly and time-consuming [2,3]. Meanwhile, alternative therapies for CIADs are gaining popularity [4]. One such approach is the use of helminths, or their products to treat CIADs. The therapeutic strategy of using helminths to subvert aggressive inflammatory immune responses in CIADs is known as helminth therapy (HT) [5]. Helminths are a group of parasitic organisms that infect humans. They are large multicellular organisms comprising nematodes, cestodes, and trematodes. Over one-sixth of the world’s population is infected with parasitic helminths [6,7]. The proposition of using HT for treating CIADs emerged in the early 1990s [5,8]. Developed countries with improved hygiene, and deworming programs, often report an increase in the incidences of CIADs, whereas, underdeveloped countries where parasitic infection is greater have fewer cases of CIADs [9,10]. Theories, such as ‘hygiene hypothesis’ and later revised as ‘old friends’ hypothesis’, proposed the symbiotic relationship between helminths and human [11,12,13]. Thus, an inverse relationship exists between childhood exposure to moderate helminth infections and the emergence of allergies later in life [12,13]. Moderate exposure to helminths was found to be beneficial to the host in reducing CIADs symptoms [10]. Subsequently, supporting evidences came from a plethora of studies, where a positive association between exposure to helminths and reduced occurrence of CIADs was clearly demonstrated [5,14,15].

The human immune system is a pool of several concatenated components involving complex mechanisms. Helminths have evolved several survival strategies to evade the human immune system, which include secreting several immunomodulatory molecules that can manipulate host inflammatory immune responses [16]. The immunosuppression induced by helminth parasites has been exploited by several laboratories around the world to suppress inflammation, allergies, and severe immune-mediated reactions [17,18]. Initial studies utilized live or attenuated parasites or their eggs to treat immune-mediated disorders [17]. However, using live parasites (including their eggs) had several aesthetic concerns [19]; (1) parasites must elicit minimum pathology in the human, especially live parasites in immuno-vulnerable patients (infants/elderly) or immunocompromised individuals could pose the risk of infection, (2) skewed immune response could expose the host to other opportunistic infections, (3) aberrant migration of the helminth parasites may cause problems, (4) the potential for worms to establish and reproduce if the infection is chronic, and (5) most importantly, ethical considerations associated with the use of live helminths. These drawbacks prevented further use of live helminth therapy. Immunologists started looking for molecules secreted by the parasites (excretory–secretory and soluble proteins) that have immunomodulatory activity. Soluble egg and worm proteins or excretory–secretory proteins from *Schistosoma mansoni*, *Ancylostoma caninum, Anisakis, Trichuris suis,* and *Brugia malayi* are shown to inhibit immune responses in experimental colitis, rheumatoid arthritis, multiple sclerosis, and type-1 diabetes [20,21,22,23,24,25,26,27,28,29]. Advances in the genomics and proteomics of helminth parasites identified several molecules secreted by the parasites that have significant host immunomodulatory activities [8]. Functional studies on these molecules demonstrated significant therapeutic potential against several CIADs [4,30]. Some of the molecules, such as ES–62, *Fasciola hepatica* FhCL1 (cathepsin L cysteine protease) and FhHDM (peptide with a cathelicidin-like C-terminal alpha helix), *S. mansoni* derived chemokine binding proteins, Sj16, Sm16, etc., have been shown to have modulatory effects on inflammation and autoimmunity [29]. In this review, we will focus on one such immunomodulatory molecule called Cystatin, which is secreted by certain helminth parasites and is believed to help the parasites evade the host immune responses and thus survive in a hostile environment [31]. Cystatin is a cysteine protease inhibitor that is most extensively studied for its immunomodulatory function in the host. 

## 2. Cystatins

**Figure 1:**
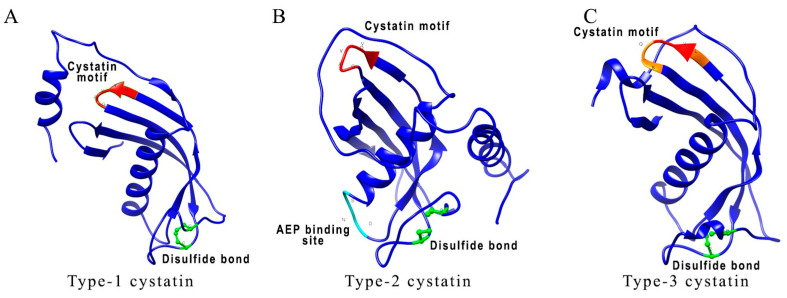
Comparison between different types of *Brugia malayi* cystatins. (**A**–**C**) 3D schematic representations of cystatin’s protein structures show conserved sites, such as QVVAG (highlighted in red), asparaginyl endopeptidase (AEP) binding loop (highlighted in cyan), and position of cysteine residues involved in disulfide bonding (highlighted in green). The proposed AEP binding loop residues are present only in *B. malayi* CPI-2. In *B. malayi* CPI-3, conserved QVVAG sites are modified to LVVQS sites (modified residues are shown in orange). Accession number for protein sequences used in structural analysis are as follows: *B. malayi* CPI-1 (AAC47623.1); CPI-2 (AAB69857.1); CPI-3 (VIO99455.1). Molecular graphics and analyses performed with UCSF Chimera, developed by the Resource for Biocomputing, Visualization, and Informatics at the University of California, San Francisco, with support from NIH P41-GM103311 [39].

Cystatins are naturally occurring intracellular cysteine protease inhibitors that are extensively expressed across all living organisms, from protozoa to mammals. Cystatins are mostly localized in the endosomes and lysosomes [32], however, they can also be found in the nucleus, cytosol, cell membrane, or secreted from the cells [33]. Cystatins participate in many vital physiologic processes and interfere in the immune processes (antigen processing and presentation, migration of immune cells, activation of toll-like receptors, and cytokines secretion), wound healing, bone remodeling, osteogenesis and reabsorption, proprotein processing, and disease progression (such as cancer and inflammation) [34,35,36,37]. Furthermore, they also participate in regulating lysosomal cathepsins and in defense mechanisms against microbial and parasitic infections [31]. The key structural characteristics of cystatins are highly conserved. X-ray crystallographic studies of cystatins revealed three highly conserved regions forming a distinct wedge-shaped structure that blocks the active site of C1 cysteine proteases [38]. Some of the conserved structural elements include N-terminal signal peptide, an approximately 100 amino acid domain, two disulfide bonds, a central glutamine–valine–glycine motif, and a C-terminal pro-trp hairpin loop (Figure 1). Some cystatins (human C, E, and F and all type-2 cystatins) also possess asparaginyl endopeptidase (AEP) inhibitory activity in addition to cysteine–protease inhibitory activity [31,38]. Based on the expression, localization, structural variations, and physiological roles, the three major families of cystatins include type-1 or stefins (cystatins A and B), type-2 (cystatins C, E, and S), and type-3 or kininogens [33]. 

Type-1 cystatins, also known as stefins, are primarily intracellular proteins with a brief appearance in body fluids [40]. Stefins are about 100 amino acid residues long, not glycosylated, and lacks disulfide bonds (Figure 1A). Stefins are present in germinal centers of secondary lymphoid organs and follicular dendritic cells (DC) [41]. Stefins have regulatory potential for exogenous proteases [42]. They also play an important role in neonatal skin development and immune responses [43]. In the presence of stefin B, IFN-γ activated mouse peritoneal cells showed enhanced release of nitric oxide (NO), suggesting a potential modulatory role of stefins on macrophages [44]. Mutations in the stefin B gene result in neurological dysfunction characterized as a form of epilepsy [45,46]. Stefin B deficiency led to the downregulation of IFN-γ regulated genes in microglia and the resident tissue macrophages in the central nervous system of mice showing their involvement in innate immune responses [47,48]. 

Type-2 cystatins are secreted as single-chain proteins of about 120 amino acids long and contain two conserved disulfide bridges and an N-terminal signal sequence (Figure 1B) [42]. There are seven different types of identified type-2 cystatins [42]. Cystatin C is one of the most abundant type-2 cystatins expressed in human tissues [33]. Cystatin C also has an additional AEP inhibitory segment located on the loop at the C-terminal end of the alpha-helix. Type-2 cystatin levels are high in body fluids acting as emergency inhibitors of redundant proteolytic activity outside cells [49] and have a diagnostic value for several diseases [50]. Cystatin C is particularly important in assessing kidney functions and is recommended by regulatory agencies for the evaluation of the glomerular filtration rate [51]. Serum levels of cystatin C in association with serum creatinine, serve as a prognosis and/or diagnostic biomarker for assessing kidney function [52]. Type-2 cystatins are shown to take part in several immune-regulatory activities [42]. Cystatin C has been shown to strongly inhibit the cathepsin S and L activity and invariant chain (li) processing in DC [53]. Nematode cystatins also come under the category of type-2 cystatins. They are shown to inhibit cathepsins B, L, S, and AEP [40]. Nematode cystatins have also been involved in regulating antigen processing and presentation in antigen-presenting cells (APC), and subsequent downmodulation of host T-cell responses [40]. This review will focus on discussing the immunoregulatory and therapeutic capabilities of nematode cystatins. 

Type-3 cystatins (kininogens) are the most complex cystatins consisting of three type 2 domains. Kininogens are about 350 amino acid residues long, mostly glycosylated, and consist of eight disulfide bonds (Figure 1C) [33]. Kininogens are secretory proteins and provide systemic protection against leaking endolysosomal cysteine peptidases. They are involved in both innate and adaptive immune responses [40,54].

## 3. Parasite Cystatins as Immunomodulators

Parasite cystatins, including other immunomodulatory molecules, are critically involved in the establishment of active parasitism in the hosts [31]. Amongst the different types of cystatins secreted by the parasites throughout their life stages, type 2 cystatins are known to significantly contribute towards immunosuppression in humans [31]. Cystatins are essentially produced by parasites to suppress host immune responses so that the parasite can survive in the host [55]. The mechanism by which parasite cystatins impairs host immune responses involves inhibition of antigen processing and presentation in APC, suppression of T cell proliferation, regulation of pattern recognition receptors, and receptor-mediated modulation of macrophages and dendritic cells by triggering the release of suppressive cytokines.

### 3.1. Inhibition of Antigen Processing and Presentation

**Figure 2:**
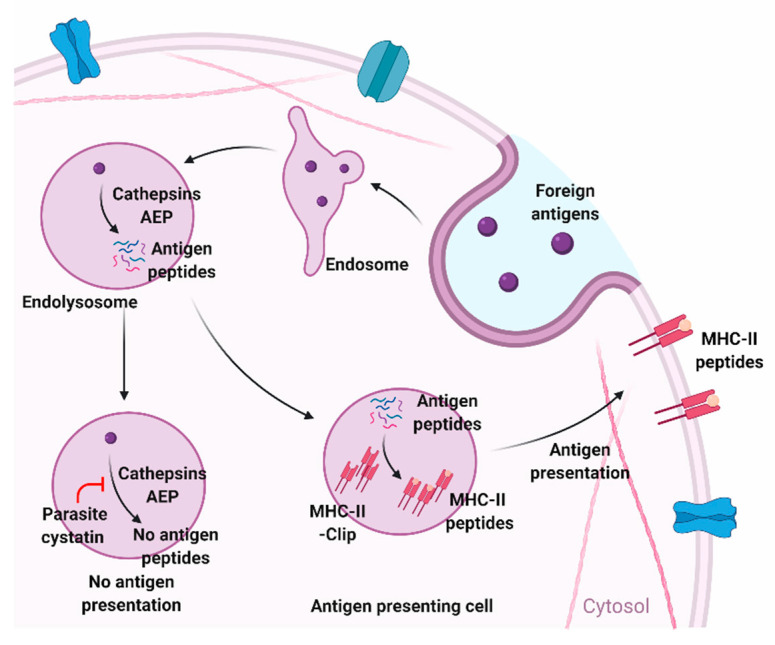
Modulation of antigen-presentation by parasite cystatins in antigen-presenting cells. Cysteine proteases and asparagine endopeptidase (AEP) degrades foreign antigens and present them on the surface of antigen-presenting cells (APC) as MHC-II-peptide complexes. Cystatin gets involved in this antigen degradation and presenting mechanism of APC by blocking cysteine proteases and AEP. Figure created using Biorender.com.

Antigenic peptides are expressed on the surface of APC, such as macrophages and dendritic cells. Cysteine proteases present in the endosomal compartments play an essential functional role in this process of antigen processing and presentation by trimming and cutting of protein antigens and cleaving off the invariant chain (Figure 2) [56]. These degraded proteins undergo epitope selection and presentation on the cell surface in the context of MHC Class II [56]. This process of antigen presentation is normally regulated by various endogenous protease inhibitors, such as cystatins that act as inhibitors for endosomal cysteine proteases. Parasite cystatins are also shown to inhibit endosomal cysteine proteases, thereby modulating immune activation of host cells (Figure 2) [31,38,55,57]. Cystatins from different parasites are shown to possess papain inhibitory enzymatic activity in vitro [38,54,58,59,60]. This inhibitory activity of cystatins can also interfere with the Class II antigen presentation system in APC by efficiently blocking cathepsin activity. Blocking antigen degradation by cystatins leads to reduced MHC-II antigen presentation in APC. Onchocystatin from the Onchocerca parasite inhibits the purified protein derivative (PPD)-specific proliferation of APC in human PBMC cultures, suggesting interference of the antigen presentation by APCs induced with parasite cystatins [61]. Recombinant cystatin from *Onchocerca volvulus*, *Ascaris lumbricoides*, and *Heligmosomoides polygyrus* can reduce the surface expression of HLA-DR, CD40, and CD86 co-stimulatory molecules on human monocytes, which are critical for antigen presentation [61,62,63,64]. Downmodulation of HLA-DR and CD86 expression by the parasite cystatins suggest an alternative receptor-mediated effect of cystatin on APC. Recombinant tick cystatin and *Trichinella spiralis* cystatin also show similar modulatory effects on APC hampering antigen presentation and downregulation of surface expression of CD80 and CD86 [65,66,67].

Few parasite cystatins, such as CPI-2 from *B. malayi* filarial parasite, also possess an additional asparginyl endopeptidase (AEP) inhibitory site that may also interfere with the MHC Class II antigen processing in human APCs by inhibiting asparginyl endopeptidase [38]. AEP inhibitory sites are common in human cystatins but not in all nematode cystatins. This feature is probably acquired by certain nematodes during their co-evolution with humans. AEP are endo or lysosomal cysteine endopeptidase that play a key role in the maturation of toll-like receptors [58]. Thus, inhibiting AEP can block toll-like receptors’ (LPS) mediated signaling pathways leading to reduced production of inflammatory cytokines and other co-stimulatory molecules [68]. 

### 3.2. Inhibition of Pattern Recognition Receptors

Cystatins may also interfere with the processing of pattern recognition receptors (PRR) [69]. PRR are key elements of the innate immune system. They are mainly expressed on the surface of macrophages, monocytes, dendritic cells, and epithelial cells acting as the first line of defense for parasite detection and capable of recognizing pathogen-associated molecular patterns (PAMPs). PRR include the toll-like receptors (TLRs), cytosolic NOD-like receptors, and the RNA-sensing retinoic acid-inducible gene (RIG)-like helicases [69]. Recognition of PAMPs by PRR leads to the activation of signaling cascades, such as the activation of the NF-κB-dependent pathway along and the interferon regulatory factor pathway, further driving the release of proinflammatory cytokines [70]. By interfering with PRR processing, cystatins might hinder the activation of APC for PAMPs [69]. 

### 3.3. Modulation of Cytokines and Nitric Oxide

**Figure 3:**
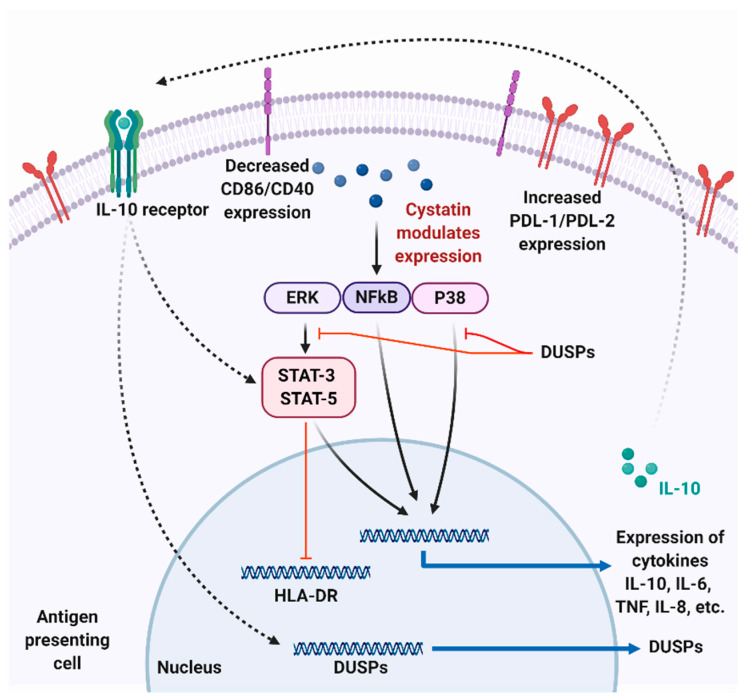
Possible mechanism of parasite cystatin mediated modulation in antigen-presenting cells. Cystatins modulate the ERK, NFkB, and P38 pathways leading to an increased expression of IL-10 and IL-8 and low expression of TNF-α and IL-6 cytokines. Secreted IL-10 can then bind to IL-10R on APC, subsequently leading to the expression of dual specific phosphatases (DUSPs) that can negatively modulate NFkB and P38 pathways. Parasite cystatins also reduce the appearance of human leukocyte antigen—DR isotype (HLA-DR), CD86, CD40, and increase expression of PDL-1 and PDL-2 in APC, which can reduce T cell activation and proliferation. Figure created using Biorender.com.

IL-10, an anti-inflammatory cytokine is responsible for optimally modulating Th1 immune responses, APCs, and T cells [71]. IL-10 limits the immunogenic response of APC by terminating excessive T-cell responses, inducing regulatory T cells, and reducing the production of proinflammatory cytokines [72]. IL-10 mediates its effects on APC through the Janus kinase 1 (JAK1)/Tyrosine kinase 2 (Tyk2)/Signal transducer and activator of transcription 3 (STAT3) pathway [73,74]. Binding of IL-10 to IL-10 receptor α (IL-10Rα) phosphorylates and activates STAT3 and STAT5 and allows its translocation to the nucleus, which then selectively modulates transcription of anti-inflammatory and inflammatory mediators [75,76]. The parasites may induce immunoregulatory effects by modulating IL-10 milieu in the host [77]. Parasite cystatins are shown to induce IL-10 secretion from macrophages and DC [59,61,78,79]. *B. malayi* cystatin has been shown to induce IL-10 in human monocytes and macrophages via the p38 dependent pathway in monocytes and also the ERK-dependent pathway in macrophages [80]. *A. viteae* cystatin also mediates IL-10 production in murine macrophages via the tyrosine kinase pathway and is dependent on the activation of mitogen-activated protein kinases (MAPK) [78,81]. The activation of MAPK ERK (extracellular-signal-regulated kinase) and p38 to induce IL-10 production has been negatively regulated by dual specific phosphatases (DUSP) [82]. Both the in-silico approach and in vivo studies showed that *A. viteae* cystatin induces the expression of DUSP1 and DUSP2 [81]. *A. viteae* cystatin also induced phospho-ERK regulated phosphorylation of STAT3 [81]. Similar effects were observed by tick cystatin on bone marrow-derived dendritic cells (BMDC) with effects on p38, ERK (MAPK), and STAT expression strengthening the evidence that cystatins can modulate APC by activating multiple pathways, as shown in Figure 3 [83].

Like IL-10, NO is another physiologic key molecule modulated by some parasite cystatins. NO is released by activated macrophages to inhibit parasitic invasion and induce host-protective immune responses, a common phenomenon against infections [59]. However, studies have also shown T-cell suppression by NO in parasitic infections [84,85,86]. This suggests a dual role played by NO in immune modulation. Cystatins from *B. malayi*, *O. volvulus*, *A. viteae,* and *Angiostrongylus cantonensis* are shown to induce NO secretion from IFN-γ primed macrophages, a response partly dependent on TNF-α and IL-10 [59,87]. The mechanism of this NO release is shown to be different from that induced by LPS in murine APC, suggesting that parasite cystatins possibly manipulate APC through a different receptor-mediated pathway. However, recombinant cystatin from *Litomosoides sigmodontis* and *S. japonicum* appears to reduce NO secretion from murine peritoneal macrophages [79,88]. This effect is different from those observed by others [59,87]. This might be due to the fact that parasite cystatins also trigger IL-10 secretion, and the timing of IL-10 expression has an ambiguous effect on NO secretion. Thus, determining the effect of parasite cystatins on NO release is complicated and can be influenced by the exposure of parasite cystatins to immune cells. This means that parasite cystatins do not induce the NO synthesis pathway, rather they seem to modulate NO production. This provides parasites an extraordinary ability to exert selective phenotypes of macrophages (M1, M2, or alternatively activated macrophages), downregulate macrophage inflammatory responses, and inhibit T-cell proliferation [89,90]. Our studies have also shown that the *B. malayi* cystatin could induce alternative activation of murine peritoneal macrophages with decreased NO and increased arginase activity [91]. However, the exact mechanism through which cystatins modulate these responses needs further investigation.

### 3.4. Suppression of T-Cell Proliferation

Filarial cystatins also showed suppression of T-cell proliferation [92]. *Acanthocheilonema viteae* cystatin can inhibit murine T cell proliferation in vitro [78]. *O. volvulus* CPI-2 reduces polyclonal and antigen-specific T cell proliferation [61,92]. *L. sigmodontis* cystatin reduced antigen-specific splenocyte responses in mice [88]. These T-cell suppressive effects of cystatins were associated with IL-10 and NO secretion from macrophages and immune cells [92]. 

## 4. Therapeutic Potential of Parasite Cystatins

Experimental studies in animal models for CIADs have provided valuable information on the beneficial effects of parasite cystatins (summarized in Table 1). Although these animal models do not exactly reflect the pathology in human disease conditions, the outcomes can be used as a guideline for developing cystatin as biotherapeutics.

### 4.1. Inflammatory Bowel Disease

Inflammatory bowel disease (IBD) is a chronic and relapsing inflammatory condition of the gastrointestinal tract. According to the Centers for disease control and prevention (CDC), about 1.3% of United States’ adults have been diagnosed with IBD. Therapeutic effects of cystatins are widely tested in various chemically induced and knockout animal models for IBD. Schnoeller et al. [93] were the first to demonstrate the therapeutic potential of a recombinant cystatin from *A. viteae* (Av17) in murine models of dextran sodium sulfate (DSS)-induced colitis. Av17-treated colitis animals showed a significant reduction in the colitis inflammatory index and also improved pathology of the colon with less inflammatory cell infiltrations. Another study using AvCystatin, highlighted the role of IL-10 from regulatory macrophages (Mregs) in the suppression of the colitis conditions [96]. Transfer of AvCystatin-Mregs to DSS-colitis mice protected animals from developing clinical signs of colitis. AvCystatin-Mregs isolated from the peritoneum of treated mice showed increased expression of M2b markers and arginase-1. This study also showed a transient expression of IL-10 in macrophages by AvCystatin uses MAPK signaling pathways. A synergistic effect of genetically modified a probiotic bacterium *E. coli* Nissle 1917 to secrete *A. viteae* cystatin has shown extraordinary effects in reducing intestinal inflammation in both mice and pigs [95].

Our group has shown the therapeutic capabilities of cystatin from filarial a parasite *B. malayi* (r*Bma*Cys) in both acute and chronic colitis. r*Bma*Cys-treatment given to DSS-induced colitis mice reduced the clinical and pathological severity of disease conditions in mice [3,99,100]. The effect was associated with an upregulated expression of IL-10 and decreased expression of IFN-γ, TNF-α, IL-5, IL-6, and IL-17 in the treated mice. Recently, we could show that IL-10 producing B1 cells in the peritoneal cavity of mice may have a possible role in promoting the immunosuppressive effects of r*Bma*Cys [91]. Further studies are required to confirm this observed therapeutic role of B1 cells. 

Cystatin from *S. japonicum* has also been widely tested against experimental colitis. rSjcystatin administration significantly ameliorated the tri-nitrobenzene sulfonic acid (TNBS)-induced colitis with increased Tregs in mesenteric lymph nodes (MLN) and lamina propria mononuclear cells (LPMC) of the treated mice [102]. The observed therapeutic effect of cystatin is believed to be through the modulation and suppression of local inflammatory responses, downregulation of Th1 immune responses, elevation of CD4+ CD25+ Foxp3+ Treg cells in the MLN, and upregulated expression of Th2 and regulatory markers IL-10, TGF-β, and Foxp3 in treated mice. There was an increase in the alternatively activated intestinal macrophages (M2-like cells) in the rSjCystatin-treated mice. A similar observation was reported by several other research groups as well [93,103,106]. Taken together, these findings suggest a more prominent role for cystatin-activated peritoneal macrophages in the disease attenuation.

Cystatins from other parasites, such as *Ascaris lumbricoides* and *Clonorchis sinensis,* have also shown potential ameliorative effects on DSS-induced colitis in experimental animals [105,106]. In mice with induced colitis, when treated with *C. sinensis* Stefin-1, the IL-10 levels were significantly increased in the spleen and MLN along with an increase in IL-10+ F4/80+ macrophages. These studies demonstrate the potential of parasite cystatin as a biotherapeutic for colitis.

### 4.2. Allergies

About 10–30% of the world’s population is affected by allergies [107]. Allergies are the most common and prominent inflammatory disorders. Surprisingly, parasite cystatins have been shown to suppress induced allergic conditions in experimental animals [18]. Treatment with *A. viteae* cystatin (Av17) in a murine model of OVA-induced allergic airway responsiveness significantly inhibited the eosinophil recruitment into the lungs of treated mice, lowered the levels of antigen-specific and total IgE, thereby reducing sensitization to mast cells and basophils [93]. A predominant role for Av17 activated macrophages in the suppression of allergic conditions and a role for T regulatory cells in the cystatin-induced suppression of inflammation and alleviation of symptoms was observed [93]. Subsequently, a single adoptive transfer of Mreg cells from *A. viteae* cystatin treated mice followed by the induction of the allergen challenge resulted in the suppression of allergen-specific IgE and reduced the infiltration of eosinophils into the lungs of mice with suppression of generalized Th2 immune responses in the lungs [96]. *A. viteae* cystatin treatment also alleviated the timothy grass pollen-induced allergic responses in a mouse model [94]. Surprisingly, increased secretion of IFN-γ was observed from CD4+ T cells in *A. viteae* cystatin treated mice. This increased IFN-γ seems to have beneficial effects in suppressing allergen-specific Th2 immune cells, a response observed in atopic patients after immunotherapy [108]. However, how *A. viteae* cystatin induces both IL-10 and IFN-γ to reduce allergic condition is not clear yet and needs further investigation. A recent study showed *A. lumbricoides* cystatin (rAl-CPI) treatment significantly reduced house dust mite-induced allergic airway inflammation [62]. After exposing human monocyte-derived DCs (HmoDCs) to rAl-CPI, the expression of HLA-DR, CD83, and CD86 was lowered with a concomitant increase in the expression of IL-10 and a decrease in the expression of IL-6 [62]. These findings demonstrate that the parasite cystatin has ameliorative effects on allergic conditions.

### 4.3. Rheumatoid Arthritis

Rheumatoid arthritis is a chronic inflammatory condition of joints. This disease affects approximately 54.4 million (25%) people in the United States and is estimated to affect over 78 million by 2040 [109]. Several studies show that both preventive and therapeutic administration of *B. malayi* cystatin could suppress the severity of mBSA-induced arthritis [97,98]. Treated animals showed a reduction in paw swelling and a reduction in clinical disease parameters accompanied by a Th2 dominant immune response [97,98]. Collagen-induced arthritis in DBA/1 mice was significantly attenuated following rSjCystatin treatment. These treated mice had upregulation of Treg cells and a skewed Th2 dominant immune response in the periphery with inhibition of Th1 and Th17 immune responses [101]. The mechanism of cystatin-induced suppression of arthritis appears to be a general suppression of RANKL (receptor activator of nuclear factor kappa-B ligand) expression in osteoclasts [97]. RANKL is essential for differentiation and activation of osteoclasts and synoviocytes [110]. Additional studies are required to establish the mechanisms by which cystatin induces its therapeutic effects in rheumatoid arthritis.

In addition to these three diseases (IBD, allergy, and rheumatoid arthritis), cystatin treatment has been found to be highly effective in other immune-mediated disorders, such as sepsis and type-1 diabetes [103,104]. SjCystatin treatment for sepsis was associated with a reduced expression of MyD88, a canonical adaptor molecule for inflammatory TLR signaling pathways in the liver, kidney, and lung tissues of the treated mice [103]. In addition, there were elevated levels of IL-10 and TGF-β1 in the sera of treated mice. This study suggests that SjCystatin could stimulate increased secretion of inhibitory cytokines from Tregs and other immune cells, which in turn can change the proinflammatory cell phenotypes. This activity of SjCystatin can be through modulation of TLR-MyD88 activation pathway in immune cells. rSjCystatin treatment also reduced the development of type-1 diabetes (up to 60%) in non-obese diabetic mice [104]. The islet cells’ pathology was conserved in treated mice with increased Tregs in spleens and PLN. Collectively, these experimental studies indicate that parasite cystatin has tremendous potential as a biotherapeutic molecule for CIADs. 

## 5. Conclusions

Immune-mediated conditions are complex, and the incidence of these disorders is overwhelmingly increasing. Better therapies and cure are urgently needed. Epidemiological and experimental evidences support the principle of helminth therapy (HT). Given the limitations of the use of HT, much attention has been recently focused on identifying specific immunomodulatory molecules of the parasites and evaluating their biotherapeutic potential in various CIADs. Among the various molecules identified, cystatins of helminth parasites are the most extensively studied immunomodulatory molecules with demonstrated activity as a biotherapeutic molecule for CIADs. Studies in various experimental animal models confirm the therapeutic potential of this molecule. Several studies attempted to identify the mechanism by which cystatin induces its immunomodulatory activity. These studies identified a role for macrophages, IL-10, regulatory cells, and several receptors and signaling molecules. Nevertheless, there is a need to identify the comprehensive mechanism by which parasite cystatin induces the generalized immunosuppression, which can be harnessed to develop this molecule further as a therapeutic molecule for treating autoimmune conditions including CIADs. 

## Figures and Tables

**Table 1:** Summary of animal experiments evaluating the therapeutic effects of parasite cystatins in immune-mediated disorders.

Cystatin Derived from Helminth Parasite	Experimental Inflammatory Condition	Outcome of the Study	Mechanism of Action	Reference
*Acanthocheilonema viteae*	OVA-induced airway inflammation DSS-induced colitis	Suppressed OVA-induced allergic airway hyperreactivity Reduced eosinophil infiltration in lungs Reduced levels of the OVA-specific and total IgE levels Suppression of DSS-induced colitis	Macrophage mediated and IL-10 dependent inhibition of the disease conditions Down-regulation of the Th2-mediated hyperresponsiveness Restricted role of Tregs	[93]
	Pollen sensitized allergy	Suppression of allergen-specific airway inflammation Reduced eosinophil infiltration, allergen-specific IgE and IL-5 and IL-13 cytokine levels in bronchoalveolar lavage	Increased IL-10 levels from splenocytes Shift of immune response towards Th1	[94]
	DSS-induced colitis	Utilized genetically modified probiotic bacterium *Escherichia coli* Nissle 1917 to secrete AvCys in the gut Decreased intestinal inflammation in mice and pigs	Increased Foxp3+ Tregs in treated mice Reduced levels of IL-6 and IL-17A, decreased macrophage inflammatory protein-1α/β and monocyte chemoattractant protein -1/3 in colon of mice	[95]
	OVA-induced airway inflammation DSS-induced colitis	Adoptive transfer of Mregs suppressed the OVA-induced airway inflammation and acute intestinal inflammation Suppressed allergen-specific IgE levels Reduced influx of eosinophils into the airways Reduced mucous production in bronchioles and lower airway cell infiltration Suppressed clinical and pathological parameters of colitis	Mregs are involved in the immunosuppressive activity of cystatin Cystatin induces conversion of classical macrophages towards a suppressive M2a/M2b hybrid phenotype Marked reduction in the Th2 cytokines along with an associated increase in the IL-10 levels	[96]
*Brugia malayi*	DSS-induced colitis	Reduced severity of the clinical disease condition Reduced damage to colon along with the restricted cellular infiltration	Reduced expression of Th1 and Th17 cytokines Possible role of Tregs and peritoneal macrophages Increased IL-10^+^ peritoneal B1 cells	[3,91]
	mBSA-induced rheumatoid arthritis	Both preventive and therapeutic suppressive effect on the RA Reduced paw swelling and cartilage destruction	Dominant Th2 immune responses with up-regulated IL-10 cytokine levels	[97,98]
	DSS-induced chronic colitis	Improved clinicopathologic condition of chronic colitis	Upregulated mRNA expression of IL-10 and TGF-β Reduced NF-κB levels in colon	[99,100]
*Schistosoma japonicum*	CIA-induced arthritis	Prevented cartilage destruction	Inhibitory modulation of Th1 and Th17 Upregulation of Tregs and Th2 shift of immune system	[101]
	TNBS-induced colitis	Reduced clinical disease parameters and ameliorated the severity of the disease condition	Decreased CD4+IFNγ+ expressing Th cell subsets in spleen, MLN and LPMC Upregulated expression of Th2 and regulatory cytokines (IL-4, IL-13, IL-10, and TGF-β) in the colon tissues Up-trending Tregs in the MLN and LPMC	[102]
	Sepsis	About 60–80% survival rate after rSj-Cys treatment Reduced disease pathology	Reduced serum levels of TNF-α, IL-6, and IL-1β cytokines	[103]
	Type-1 diabetes; NOD mice	60% reduction in diabetes incidence Preserved islet cells pathology in treated animals	Increased Treg cells Reduced IFN-γ and increased IL-4, IL-10, and TGF-β levels in spleens and PLN	[104]
*Ascaris lumbricoides*	DSS-induced colitis	Inhibition of the disease condition Significant reduction in disease activity score, myeloperoxidase activity and histopathological damage of colon	IL-10 and TGF-B gene overexpression in rAl-CPI treated colitis mice	[105]
	HDM-induced allergic airway inflammation	Significantly reduced allergy symptoms in treated mice Reduced cellular infiltration in BAL	Decreased allergy specific Th2 immune response Decreased total and IgE specific antibodies Increased Tregs in spleen and IL-10 in BAL and spleen HmoDCs with lowered expression of HLA-DR, CD83, and CD86	[62]
*Clonorchis sinensis*	DSS-induced colitis	Curative treatment lead to the amelioration of the disease condition		[106]

OVA: ovalbumin; DSS: dextan sulfate sodium; RA: rheumatoid arthritis; mBSA: methylated bovine serum albumin; TNBS: tri-nitrobenzene sulfonic acid; CIA: collagen-induced arthritis; MLN: mysenteric lymph nodes; LPMC: lamina propria mononuclear cells; NOD: non-obese diabetic; PLN: peripheral lymph nodes; HDM: house dust mite; BAL: bronchoalveolar lavage; HmoDCs: human monocyte-derived dendritic cells; HLA-DR: human leukocyte antigen—DR isotype.
